# Phylogenetic and Molecular Analyses of More Prevalent HCV1b Subtype in the Calabria Region, Southern Italy

**DOI:** 10.3390/jcm10081655

**Published:** 2021-04-13

**Authors:** Nadia Marascio, Angela Costantino, Stefania Taffon, Alessandra Lo Presti, Michele Equestre, Roberto Bruni, Giulio Pisani, Giorgio Settimo Barreca, Angela Quirino, Enrico Maria Trecarichi, Chiara Costa, Maria Mazzitelli, Francesca Serapide, Giovanni Matera, Carlo Torti, Maria Carla Liberto, Anna Rita Ciccaglione

**Affiliations:** 1 Department of Health Sciences, Institute of Microbiology, “Magna Grecia” University, 88100 Catanzaro, Italy; gbarreca@unicz.it (G.S.B.); quirino@unicz.it (A.Q.); gm4106@gmail.com (G.M.); mliberto@unicz.it (M.C.L.); 2 Department of Infectious Diseases, Istituto Superiore di Sanità, 00161 Rome, Italy; angela.costantino@iss.it (A.C.); stefania.taffon@iss.it (S.T.); alessandra.lopresti@iss.it (A.L.P.); roberto.bruni@iss.it (R.B.); annarita.ciccaglione@iss.it (A.R.C.); 3 Department of Cell Biology and Neuroscience, Istituto Superiore di Sanità, 00161 Rome, Italy; michele.equestre@iss.it; 4 National Center for Immunobiologicals Research and Evaluation, Istituto Superiore di Sanità, 00161 Rome, Italy; giulio.pisani@iss.it; 5 Department of Medical and Surgical Sciences, Unit of Infectious and Tropical Diseases, “Magna Graecia” University, 88100 Catanzaro, Italy; em.trecarichi@unicz.it (E.M.T.); c.costa@materdominiaou.it (C.C.); m.mazzitelli88@gmail.com (M.M.); francescaserapide@gmail.com (F.S.); torti@unicz.it (C.T.)

**Keywords:** hepatitis C virus (HCV), phylogeny, resistance-associated substitution (RAS)

## Abstract

Hepatitis C virus subtype 1b (HCV1b) is still the most prevalent subtype worldwide, with massive expansion due to poor health care standards, such as blood transfusion and iatrogenic procedures. Despite safe and effective new direct antiviral agents (DAA), treatment success can depend on resistance-associated substitutions (RASs) carried in target genomic regions. Herein we investigated transmission clusters and RASs among isolates from HCV1b positive subjects in the Calabria Region. Forty-one NS5B and twenty-two NS5A sequences were obtained by Sanger sequencing. Phylogenetic analysis was performed using the maximum likelihood method and resistance substitutions were analyzed with the Geno2pheno tool. Phylogenetic analysis showed sixteen statistically supported clusters, with twelve containing Italian sequences mixed with foreign HCV1b isolates and four monophyletic clusters including only sequences from Calabria. Interestingly, HCV1b spread has been maintained by sporadic infections in geographically limited areas and by dental treatment or surgical intervention in the metropolitan area. The L159F NS5B RAS was found in 15 isolates and in particular 8/15 also showed the C316N substitution. The Y93H and L31M NS5A RASs were detected in three and one isolates, respectively. The A92T NS5A RAS was found in one isolate. Overall, frequencies of detected NS5B and NS5A RASs were 36.6% and 22.7%, respectively. For the eradication of infection, improved screening policies should be considered and the prevalence of natural RASs carried on viral strains.

## 1. Introduction

Hepatitis C (HCV) infection remains a major public health problem, even if in the last few years HCV therapy has been improved by the availability of direct-acting antiviral (DAA) agents [1]. Phylogenetic analyses have identified eight HCV major genotypes, further subdivided into 67 subtypes [2]. HCV1b is widespread all over the world, HCV2 showed higher prevalence in Russia and in Italy. In Europe, the most common HCV2 subtypes are HCV2a/2c. HCV1a and HCV3a predominate in Europe and North America, while HCV4 is endemic in the Middle East, Central Africa and Mediterranean countries. HCV5 is endemic in South Africa, HCV6 in South East Asia and HCV7 was found in the Democratic Republic of Congo. Recently, HCV8 was found in Indian patients living in Canada [2,3,4]. Magiorkinis and colleagues reported a massive expansion of HCV1b infections between 1940 and 1980, sustained by blood transfusion and iatrogenic procedures [5]. In Europe, HCV1b was predominantly found in females and associated with births not later than 1958 [4]. Its prevalence is decreasing due to improved health standards [6]. Interestingly, HCV1b was predominant in Japan, Italy, and Spain with a high prevalence in patients with hepatocellular carcinoma [7]. Since 1997, HCV1b has been the most prevalent subtype in the Calabria Region reflecting national data [3,8].

The major prevalence worldwide and the low susceptibility to Interferon (IFN) or pegylated-IFN alfa with ribavirin (pegIFN-α/RBV) therapies made HCV1b the first target for the development of new antiviral drugs [9]. Currently, direct-acting-antiviral (DAA) pan-genotypic therapy can be used to treat infected people without the need for determining the genotype/subtype or performing a resistance test [1]. Pretreatment assessment should consider the presence of cirrhosis and comorbidity in view of post-therapy follow-up. However, after considering the data of DAA efficacy in a clinical setting, combination therapy still appears to be influenced by resistance-associated substitutions (RASs) carried in target regions in naïve or experienced patients [10].

In this study, we investigated transmission clusters in two cohorts of HCV1b positive subjects, enrolled in different time spans, to assess the dynamics of infection in the Calabria Region, southern Italy. In particular, more recent isolates were evaluated for the presence of mutations with a potential impact on treatment response.

## 2. Materials and Methods

### 2.1. Study Population

The study was approved by the Ethical Committee (#100; 27 April 2017) of the “Mater Domini” University Teaching Hospital of Catanzaro, Italy and it was included in the SINERGIE study [11]. The Ethical Committee approved the criteria that there is no need for informed consent for a non-interventional study. Forty-one serum samples, collected between 1 January 2015 and 31 December 2016, from patients infected by HCV subtype 1b were included in the analysis. Enrolled patients, attending the University Hospital of Catanzaro, were randomly selected from a list through a systematic 1:7 sampling procedure. The selected sample is representative of the whole HCV1b cohort, including 41.7% of males versus 54.0% of females with an overall median age 68 (31–84) years [12]. Patients were naïve to all treatments (25/41) or treated with IFN (3/41) and pegIFN-α/RBV (13/41). Additionally, only viral isolates from HCV1b positive subjects, collected between May and October 2010 during a previous epidemiological study in Calabria, were included in order to compare and investigate phylogenetic relationships with those from Catanzaro. All participants were resident in a small village, Sersale (Catanzaro province) [13]. The patients’ clinical data was treated in accordance with the Helsinki Declaration (59^th^ World Medical Association General Assembly, Seoul, Korea, October 2008) and the principles of good clinical practice.

### 2.2. Diagnostic Procedures

HCV RNA viral load was determined using the Cobas AmpliPrep/Cobas TaqMan HCV quantitative test v2.0 (Roche Diagnostics, Milan, Italy). Genotyping was performed by the Versant HCV genotype v2.0 assay (LiPA) (Siemens, Healthcare Diagnostic Inc., Tarrytown, NY, USA). Fibrosis stage was estimated by transient elastometry (FibroScan, Echosens, Paris, France), interpreted as follows: F0–F1 = minimal fibrosis (KPa ≤ 7.1), F2 = moderate fibrosis (7.1 < KPa ≤ 9.5), F3 = severe fibrosis (9.5 < KPa ≤ 14.5), F4 = cirrhosis (KPa > 14.5) [14].

### 2.3. Amplification and Sequencing of HCV NS5B and NS5A Regions

Viral RNA was extracted from 140 μL serum samples using the QIAamp Viral RNA Extraction Kit (Qiagen, Hilden, Germany) in accordance with the manufacturer’s instructions. Healthy donor serum samples were used as a negative control. The RNA was reverse transcribed using the High-Capacity cDNA Reverse Transcription Kit protocol (Thermo Fischer Scientific, Waltham, MA, USA) and cDNA amplified by nested PCR using the FastStart High Fidelity PCR system (Roche Diagnostics, Basel, Switzerland). The specific primers used to amplify the NS5B (nt 8256–8632) and NS5A (nt 6086–6722) regions of HCV genome for the first and second rounds have been previously described [15,16]. The products were purified using the High Pure PCR Cleanup Micro Kit (Roche Diagnostics, Basel, Switzerland) and analyzed on 2% agarose gel stained with GelRed (Biotium Corporete Headquarters, Biotium Inc., Fremont, CA, USA). Both strands were sequenced using the Genome Lab DTCS Quick Start KiT (Beckman Coulter, Inc., Fullerton, CA, USA). Sequencing reactions were run on an automated DNA sequencer (Beckman Coulter, Inc., Fullerton, CA, USA). HCV sequences were aligned by MAFFT under the Galaxy platform (https://usegalaxy.org/, accessed on 27 March 2020) and manually edited by using Bioedit [17,18,19].

### 2.4. Subtyping Tool Analysis

NS5B and NS5A sequences were analyzed using the Oxford HCV Automated Subtyping Tool v.2.0 (http://dbpartners.stanford.edu/RegaSubtyping/html/subtypinghcvSUB.html, accessed on 20 April 2020) and COMET HCV typing tool (https://comet.lih.lu/index.php?cat=hcv, accessed on 20 April 2020) followed by phylogenetic analysis (see below) to confirm the initial subtyping assignment by LiPA assay [20,21].

### 2.5. Datasets Construction

Two datasets were built. The first dataset contained 78 total sequences: 53 HCV NS5B new sequences from Italy (41 from Catanzaro University Hospital and 12 from Sersale) plus 25 HCV NS5B subtype specific reference sequences downloaded from the HCV Los Alamos sequence database (http://hcv.lanl.gov/content/index, accessed on 11 May 2020). The second dataset comprised 162 total sequences including: 53 HCV NS5B sequences from Italy, previously classified as 1b subtype, plus 109 foreign HCV 1b NS5B sequences downloaded from the HCV Los Alamos sequence database (http://hcv.lanl.gov/content/index, accessed on 11 May 2020).

### 2.6. Likelihood Mapping

The phylogenetic signal of each sequence dataset was investigated by means of the likelihood mapping analysis of 10,000 random quartets generated using TreePuzzle [22]. Groups of four randomly chosen sequences (quartets) were evaluated. For each quartet, the three possible unrooted trees were reconstructed using the maximum likelihood approach under the selected substitution model. Posterior probabilities of each tree were then plotted on a triangular surface so that fully resolved trees fell into the corners and the unresolved quartets in the center of the triangle (star-like trees). When using this strategy, if more than 30% of the dots fall into the center of the triangle, the data is considered unreliable for the purposes of phylogenetic inference.

### 2.7. Phylogenetic Analysis

Sequences of all datasets were aligned using MAFFT under the Galaxy platform and manually edited using Bioedit [17,18,19]. The subtypes of the newly generated sequences from Calabria were determined and confirmed by phylogenetic analysis of the first dataset. The maximum likelihood phylogenetic trees of the first and second dataset together with the estimation of the best-fit substitution models (TPM2 + F + I + G4 and TVMe + I + G4 for the first and second dataset, respectively) were performed through IQ-TREE with the Model Finder option and visualized with FigTree v. 1.4.4 [23]. Statistical support for internal branches of the maximum likelihood (ML) trees were evaluated by bootstrap analysis (1000 replicates) and fast likelihood-based sh-like probability (SH-aLRT).

### 2.8. Genetic Variability Analysis

HCV1b viral population for each patient was screened for genetic variation with a cut-off of 15% [1]. Forty-one NS5B and twenty-two NS5A sequences at specific nucleotide positions were analyzed. Non-synonymous and resistance-associated substitutions (RAS) were determined using the Geno2pheno (HCV) 0.92 tool (last updated: June 2019) and aligning generated sequences to HCV1b (AJ238799) reference by MAFFT [17,24]. Resistance prediction rules available in the online tool were implemented by literature search [25].

### 2.9. Public Availability of the Sequencing Data

The 41 NS5B and 22 NS5A newly generated sequences were submitted to the GenBank database [26]. All sequences can be retrieved from GenBank under accession numbers: MW357752-MW357814.

## 3. Results

### 3.1. Patient Demographic Characteristics and Risk Factors

The median age of the 53 patients was 70 years (range 31–90), with 58.5% females. Overall, dental treatment and surgical intervention were the first (16.9%) and second (13.2%) most frequent risk factors, followed by blood transfusion (3.8%) and cohabitation (1.9%). Only one patient reported intravenous drug use as a risk factor. Three patients declared no risk factors. Qualitative characteristics of the two cohorts are reported separately (Table 1).

### 3.2. Likelihood Mapping

The phylogenetic noise of each dataset was investigated by means of likelihood mapping (Figure S1). The percentage of dots falling in the central area of the triangles was 13.2% and 7.5% for the first and second datasets, respectively. As none of the datasets showed more than 30% of noise, all of them contained sufficient phylogenetic signal.

### 3.3. Phylogenetic Analysis

**Figure 1:**
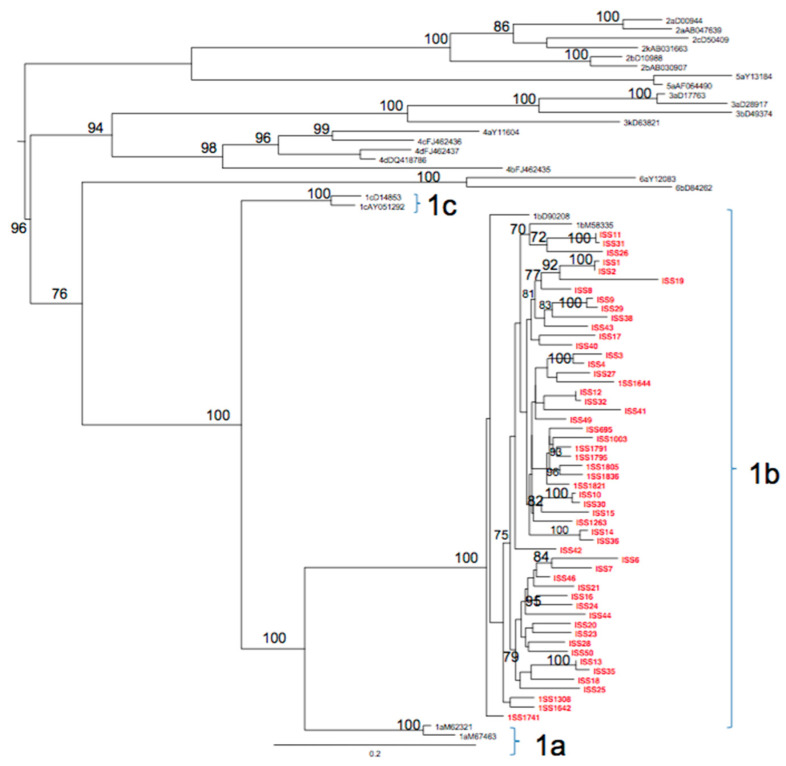
Maximum likelihood phylogenetic tree of the first HCV NS5B dataset. The tree was rooted by using the midpoint rooting method. Branch lengths were estimated with the best fitting nucleotide substitution model according to a hierarchical likelihood ratio test, and were drawn to scale with the bar at the bottom indicating 0.2 nucleotide substitutions per site. The values along a branch represent significant statistical support for the clade subtending that branch (bootstrap support >75%). The Italian (Calabria Region) sequences are highlighted in red. Clades 1b, 1c and 1a are also highlighted with brackets.

All new sequences were classified as subtype 1b by both Oxford and COMET subtyping tools, and by phylogenetic analysis. The ML phylogenetic tree of the first dataset showed that all the 53 sequences collected from the Calabria Region were in the same statistically supported clade, closely related to the references subtype 1b and were therefore classified as subtype 1b (Figure 1).

**Figure 2:**
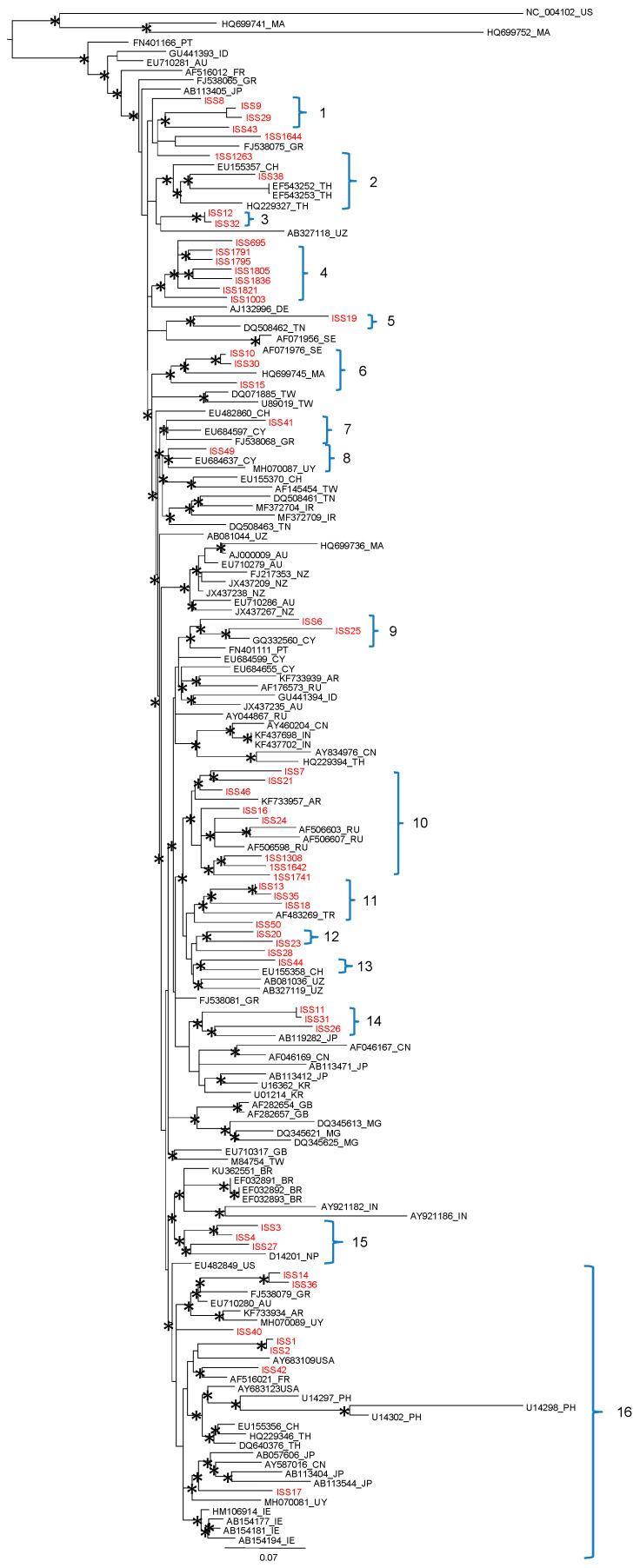
Maximum likelihood phylogenetic tree of the second dataset HCV1b dataset. The tree was rooted by using the midpoint rooting method. Branch lengths were estimated with the best fitting nucleotide substitution model according to a hierarchical likelihood ratio test, and were drawn to scale with the bar at the bottom indicating 0.07 nucleotide substitutions per site. The asterisk (*) along a branch represents significant statistical support for the clade subtending that branch (bootstrap support >75%). The main statistically significant sequences are highlighted with brackets.

The maximum likelihood phylogenetic tree of the second dataset showed the presence of a supported cluster and a main statistically supported clade (Figure 2).

The supported cluster included three foreign (Morocco and US) related sequences. Within the main clade, the HCV1b Italian (Calabria Region) sequences were distributed in 16 statistically supported clusters. Twelve clusters (12/16, 75%), presented foreign HCV 1b reference sequences intermixed with sequences from Italy (Clusters: 2, 5, 6, 7, 8, 9, 10, 11, 13, 14, 15, 16). Four statistically significant monophyletic clusters, including only sequences from Calabria were also observed (clusters: 1, 3, 4, 12).

Cluster 1 included three sequences from Catanzaro (ISS 9, 29, 43) reporting the following risk factors: blood transfusion/cohabitation, surgery/dental treatment and dental treatment, respectively.

The sequence ISS38 was located in cluster 2 with one reference from Switzerland and three from Thailand. Cluster 3 was composed of two Calabrian sequences (ISS 12 and 32) characterized respectively, by the following risk factors: dental treatment and blood transfusion/dental treatment. Interestingly, cluster 4 included seven sequences (ISS 695; 1791, 1795, 1805, 1836, 1821, 1003), closely related to each other, all from Sersale village. The following risk factors were reported: surgery, cohabitation with HCV positive, sharing glass syringes and blood transfusion (ISS 695), surgery and sharing glass syringes (ISS 1003, 1805, 1821, 1836), no risk factors (ISS 1791, 1795). Cluster 5 included one isolate from Calabria (Catanzaro) reporting blood transfusion as a risk factor and related to a sequence from Tunisia. Cluster 6 was composed of three isolates (ISS10, 30 and 15) from Catanzaro (risk factors: blood transfusion, surgery/dental treatment and surgery) related to a sequence from Morocco. Cluster 7 included one isolate (ISS 41) from Catanzaro characterized by the following risk factor: surgery, related to one reference from Cyprus and one from Greece. Cluster 8 included isolate ISS49 (risk factors: surgery and blood transfusion), one reference from Cyprus and one from Uruguay. Cluster 9 included two isolates from Catanzaro, ISS6 and ISS25 (risk factors: surgery/multiple blood transfusion and surgery/dental treatment, respectively) related to a sequence from Cyprus and another from Portugal. Cluster 10 included eight isolates from Calabria (ISS 7, 21, 46, 16, 24, 1308, 164, 1741), three of which from Sersale, related to one reference from Argentina and three from Russia. Cluster 11 was characterized by three sequences from Catanzaro (ISS 13, 35 and 18) reporting the following risk factors (surgery, surgery/dental treatment, dental treatment, respectively) and related to a reference from Turkey. Cluster 12 included two isolates collected from Catanzaro (ISS 20 and 23) with risk factors: surgery/blood transfusion and surgery/dental treatment, respectively. Cluster 13 was composed of two sequences, the isolate ISS 44 from Catanzaro (reporting surgery/cohabitation as risk factors) related to a reference from Switzerland. Cluster 14 included three isolates from Catanzaro (ISS 11, 31 and 26) reporting the following risk factors (dental treatment, surgery, blood transfusion, and cohabitation) related to a reference from Japan. Cluster 15 included three sequences (ISS 3, 4 and 27) characterized by the following risk factors: surgery/dental treatment; surgery/blood transfusion; surgery/dental treatment/cohabitation and related to a reference from Nepal. Cluster 16 included seven isolates from Catanzaro (ISS 14, 36, 40, 1, 2, 42, 17) intermixed with many sequences sampled from different countries: USA, Greece, Austria, Argentina, Uruguay, France, Philippines, Switzerland, Thailand, Japan, China and Ireland.

### 3.4. Substitutions on Target Regions in Patients Naïve to DAA

The total (100%) of NS5B amplicons were sequenced. Nine (40%) NS5A amplicons were not successfully sequenced, while 10 sequences were not of suitable length for RAS screening. Available NS5A and NS5B sequences at the time of genotyping were screened for RASs and nonsynonymous substitutions. We identified the L159F NS5B substitution, conferring resistance to sofosbuvir (SOF), in 15/41 (ISS 6, 7, 13, 16, 18, 20, 21, 23, 24, 25, 28, 35, 44, 46, 50) isolates, among them 8/15 (ISS 6, 13, 21, 24, 25, 28, 35, 46) also carried the C316N NS5B related to dasabuvir resistance. In particular, frequency of detected NS5B RASs was 36.6%, while frequency of RASs carried on NS5A region was 22.7%.

The Y93H, associated with resistance to daclatasvir, elbasvir, ledipasvir (LPV), ombitasvir (OMV) and pibrentasvir was detected on NS5A in 3/22 (ISS 16, 24, 30) isolates. The L31M substitution associated with resistance to all drugs mentioned above plus velpatasvir was found in 1/22 (ISS 21) isolate. Interestingly, all three isolates carried Y93H plus K108R substitution. The A92T NS5A OMV and LPV associated resistance was detected in 1/22 (ISS 2) isolate. Among patients who reported RASs in the viral population, seven have been previously treated with an IFN regimen with or without RBV and were classified as non-responders (4/7) or relapsers (3/7) with liver stiffness F3 or F4. On the other hand, the 33.3% of patients without RASs were IFN experienced with or without RBV. The median baseline RNA viral load was 2,280,000 IU/mL.

## 4. Discussion

In order to explore the spread of HCV1b in the Calabria Region, we analyzed NS5B population sequences, obtained from two cohorts of positive individuals, enrolled in different time spans, using phylogenetic analysis. In addition, viral isolates collected between 2015 and 2016 from naïve and IFN/pegIFN-α/RBV treated patients were analyzed in the NS5B and NS5A regions to assess the presence of RASs with the potential to impact on DAA therapy.

Molecular analysis was carried out on 53 sequences of HCV subtype 1b, previously characterized by Inno-Lipa and confirmed by sequencing analysis. As reported in previous studies, subtype 1b, together with subtype 2c, are the most prevalent genotypes in Italy followed by genotypes 3 and 4 [3,6]. HCV1b diffusion worldwide is related to several risk factors, such as blood transfusions, dental treatment, unsafe reuse of nondisposable syringes [27,28]. In previous studies, transmission of two subtypes was already correlated to specific risk factors in the Calabria Region. HCV4d was found related to intravenous drug use and blood transfusion, while HCV2c infection was maintained by unsafe use of glass syringes followed by surgery and unsafe blood transfusion [29,30].

In this work, we investigated possible transmission patterns in a regionally representative sample from a small village (Sersale), where a seminal HCV prevalence study was conducted, and a metropolitan area of the Calabria region [13]. The ML phylogenetic tree shows that the HCV1b Calabria sequences were distributed in 16 statistically supported clusters. Twelve clusters (75%), contained Italian sequences mixed with foreign HCV1b references while four statistically significant monophyletic clusters included only sequences from Calabria (clusters: 1, 3, 4, 12). In particular, cluster 4 contained only seven closely related Italian sequences collected from Sersale village.

In this study, the majority (58.5%) of the enrolled individuals reported multiple risk factors, most of which were surgical intervention and dental treatment (*n* = 8) or surgical intervention and glass syringes (*n* = 7). Individually, we observed that the most frequent risk factors were dental treatment (16.9%) and surgical intervention (13.2%). Interestingly, the risk factors for HCV acquisition in cluster 4 were medical interventions and multiple use of glass syringes in a family setting as reported in 71% (no. 5/7) of patients (ISS 695, 1003, 1805, 1821, 1836).

Our analysis indicates that in the past, subtype 1b was maintained, by sporadic infections, mainly acquired through unsafe use of glass syringes especially in some limited areas of southern Italy, such as Sersale, a small town located 30 miles from Catanzaro. Conversely, in the metropolitan area, other transmission routes, such as dental treatment and surgical intervention had a significant influence on the dissemination of HCV subtype 1b throughout the Calabria Region. Interestingly, a community-based survey in the Calabria Region, revealed a high percentage of possible risk factors for HCV acquisition, such as dental treatment (69.5%) and glass syringes injections (25.8%) [31].

On the other hand, DAA treatment of hepatitis C could be influenced by baseline RASs naturally occurring in the viral genome [25,32]. It has been reported that 3% of HCV positive patients have no virological response, due to the presence of comorbidities and/or RASs in viral isolates, especially in the NS5A viral region [33]. We detected NS5B L159F alone in 15/41 (36.5%) and in association with C316N in 8/41 (19.5%) patients, respectively. This last substitution, showing a global frequency of 31.4% in HCV1b, is now defined as a fitness-associated substitution when combined with the L159F [34]. Therefore, both amino acid variants were associated with a lower response to SOF [35]. Interestingly, NS5B S282T conferring high-level resistance to SOF-containing regimens, was not detected among our isolates, despite being present in 99.1% of worldwide strains [25]. In three patients, NS5A sequences carried the Y93H substitution, currently the major clinically relevant RAS contributing to failure of many approved IFN-free regimens [36]. Additionally, all three isolates showed the Y93H + K108R profile, which is associated with a minor affinity to OMV drug with respect to the Y93H + R108K combination as previously reported [37]. However, the 97% of treated patients with DAAs achieved sustained virological response (SVR). According to our experience about a single-center cohort in Southern Italy, the SVR rate was 97% for the older age group, 96% for people under 65 years old, finally 94% and 100% for cirrhotic and non-cirrhotic patients, respectively [38].

## 5. Conclusions

Despite the sample size being a limitation of the study, this suggests that the spread of HCV1b was maintained in the Calabria Region by sporadic infections, mainly acquired through the unsafe use of glass syringes, dental treatment and surgical intervention. Even if our analysis was performed on samples collected in 2015–2016, the frequency of natural RASs carried on subtype-specific viral strains, as well as comorbidities of treated patients, should be taken into account for the effectiveness of IFN-free regimens to eradicate HCV infection.

## Figures and Tables

**Table 1:** Patient demographic characteristics.

	Absolute Number (%)		
Characteristics	Overall	Patients from University Hospital	Subjects from Sersale Village
**Gender**			
M	22 (41.5)	21 (48.7)	1 (8.3)
F	31 (58.5)	20 (51.3)	11 (91.7)
**Risk factors**			
Surgical intervention	7 (13.2)	7 (17.1)	-
Blood transfusion	2 (3.8)	2 (4.8)	-
Dental treatment therapy	9 (16.9)	9 (21.9)	-
Cohabitation	1 (1.9)	1 (2.4)	-
Multiple *	31 (58.5)	22 (53.6)	9 (75)
Not available	3 (5.7)	-	3 (25)
**Clinical parameters**			
cirrhotic status	-	14 (34.1)	not available
HCV RNA median level	3,792,576 IU/mL	2,280,000 IU/mL	3,918,625 IU/mL
**Median (range)**			
Age (years)	70 (31–90)	68 (31–85)	71 (65–90)
**Total**	**53**	**41**	**12**

* Multiple risk factors were: surgical intervention + blood transfusion (*n* = 4), surgical intervention + blood transfusion + cohabitation (*n* = 2), blood transfusion + cohabitation (*n* = 1), blood transfusion + dental treatment (*n* = 2), dental treatment + cohabitation (*n* = 1), surgical intervention + cohabitation (*n* = 2), surgical intervention + dental treatment (*n* = 8), surgical intervention + drug user (*n* = 1), surgical intervention + dental treatment + cohabitation (*n* = 1), surgical intervention + glass syringes (*n* = 7), surgical intervention + cohabitation + blood transfusion + glass syringes (*n* = 1), cohabitation + glass syringes (*n* = 1). Characteristics heading and total number of patients were in bold.

## Data Availability

The 41 NS5B and 22 NS5A newly generated sequences used for molecular analysis have been uploaded to GenBank under the following accession numbers: MW357752-MW357814. Materials supporting the findings of this study are available within the article.

## Supplementary Materials

The following are available online at https://www.mdpi.com/article/10.3390/jcm10081655/s1, Figure S1: Likelihood mapping of HCV NS5B first (a) and second (b) dataset.
