# Supplementary material for: Phylogenetic and Molecular Analyses of More Prevalent HCV1b Subtype in the Calabria Region, Southern Italy
Source: J Clin Med. 2021 Apr 13;10(8):1655. doi: 10.3390/jcm10081655 (PMC8068798; doi:10.3390/jcm10081655)
Supplement: Supplementary file 1 [file jcm-10-01655-s001.pdf]

Phylogenetic and molecular analyses of more prevalent HCV1b subtype in the Calabria Region, Southern Italy.

Nadia Marascio[*1a], Angela Costantino[2a], Stefania Taffon[2], Alessandra Lo Presti[2], Michele Equestre[3], Roberto Bruni[2], Giulio Pisani[4], Giorgio Settimo Barreca[1], Angela Quirino[1], Enrico Maria Trecarichi[5], Chiara Costa[5], Maria Mazzitelli[5], Francesca Serapide[5], Giovanni Matera[1], Carlo Torti[5], Maria Carla Liberto[1], Anna Rita Ciccaglione[2].

[1] Department of Health Sciences, Institute of Microbiology, "Magna Grecia" University of Catanzaro, Catanzaro, Italy.

[2] Department of Infectious, Parasitic and Immune-Mediated Diseases, Istituto Superiore di Sanità, Rome, Italy.

[3] Department of Cell Biology and Neuroscience, Department of Cell Biology and Neuroscience

[4] Center for Immunobiologicals Research and Evaluation, Istituto Superiore di Sanità, Rome, Italy

[5] Department of Medical and Surgical Sciences, Unit of Infectious and Tropical Diseases, Magna Graecia University, Catanzaro, Italy.

*****Correspondence: Nadia Marascio, PhD, "Magna Graecia" University, Viale Europa – Loc. Germaneto, 88100 Catanzaro, Italy. E-mail: nadiamarascio@gmail.com, phone: +39-09613697742, fax: +39-09613697760.

a: contributed equally

Figure legend

Figure S1. Likelihood mapping of HCV NS5B first (a) and second (b) dataset. The dots inside the triangles represent the posterior probabilities of the possible unrooted topologies for each quartet. Numbers indicate the percentage of dots in the centre of the triangle corresponding to phylogenetic noise (star-like trees).

a) b)

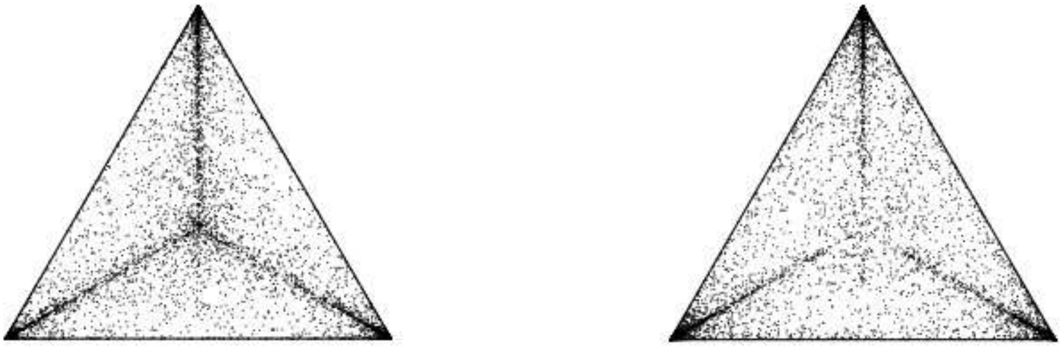

13.2% 7.5 %
